# Reflective functioning in anorexia nervosa: does it differ from healthy controls and what is its relation to psychopathology?

**DOI:** 10.1186/s40337-025-01465-x

**Published:** 2025-12-01

**Authors:** Lara Maria Kanstinger, Almut Zeeck, Armin Hartmann, Anne Marie Eyschen, Sylke Andreas, Lotta Hüwe, Claas Lahmann, Inga Lau

**Affiliations:** 1 Department of Psychosomatic Medicine und Psychotherapy, Medical Center and Faculty of Medicine, University of Freiburg, Freiburg, Germany; 2 Department of Clinical Psychology, Psychotherapy and Psychoanalysis, University of Klagenfurt, Klagenfurt am Wörthersee, Austria

**Keywords:** Anorexia nervosa, Reflective functioning, Mentalizing, Psychopathology, BRFI

## Abstract

**Background:**

Previous studies found an impairment in the capacity to mentalize (operationalized as “*reflective functioning*”, RF) in patients with anorexia nervosa (AN), but only few studies used a validated interview procedure. The aim of this study was an assessment of RF in patients with anorexia nervosa in comparison to healthy controls (HCs), using both an expert-rated measure as well as a self-report measure. Further, the study aimed to explore the relationship between RF and various aspects of psychopathology.

**Methods:**

30 female patients with AN (mean age: 26.2 ± 10.1 years) and 30 matched HCs (mean age: 27.4 ± 9.9 years) were assessed and compared regarding their level of RF using the Brief Reflective Functioning Interview (BRFI) and the Mentalization Questionnaire (MZQ). The interrelations between RF values and eating disorder psychopathology (BMI, EDI, EDE-Q), body experience (DKB-35), general psychopathology (PHQ-9, PHQ-15, GAD-7) and impairment in personality functioning (OPD-SQS) were explored using correlation coefficients.

**Results:**

On the RF-Scale (RF-BRFI), RF was M = 3.63 (SD = 0.67) in the AN group and M = 4.13 (SD = 0.94) in the HC group. It was significantly lower for patients with AN than for HCs (t(52.5) = 2.38; *p* = 0.011). Mentalizing in the AN group as assessed with the MZQ was M = 3.24 (SD = 0.71), demonstrating significantly worse self-reported mentalizing capacities compared to HCs (M = 1.73; SD = 0.45; *p* < 0.0001). RF as measured with the BRFI was only correlated to the EDI-subscale “weight concern”, while RF values of the MZQ were correlated with various aspects of psychopathology except depressive symptoms and BMI.

**Conclusion:**

The study confirmed the finding that patients with AN show impaired mentalizing. Strengthening the ability to mentalize might therefore be a relevant focus in treatment. However, associations of RF with psychopathology depend on the RF-measure used. Therefore, a better understanding of which aspects of mentalizing are captured by the RF-measures available would be an important basis for further research.

*Trial registration* DRKS00031108

## Abstract (plain language summary)

The study examined how well people with anorexia nervosa (AN) are able understand their own and others’ thoughts and feelings (“mentalizing”), compared to people without an eating disorder (ED). Both an interview rated by experts and a questionnaire completed by the participants were used. The study also examined how the ability to mentalize is related to various psychological problems such as eating disorder symptoms, body image issues and general psychological distress. A total of 30 people with AN and 30 healthy individuals participated in the study. It was found that mentalizing is more impaired in people with AN. The expert-ratings showed little relation to eating disoder symptoms or other psychological problems, while the results of the questionnaire were clearly related to several mental health difficulties. In summary, the difficulties experienced by participants with AN in mentalizing support the idea of addressing mentalizing abilities in therapy. However, the relationship between psychological problems and mentalizing capacities differed depending on how the latter were measured. Therefore, observer-rated and self-reported measures might cover different aspects of how people reflect on their own minds and feelings, as well as on those of others.

## Background

Anorexia nervosa (AN) is a severe illness with one of the highest mortality rates among all mental disorders [1], a wide range of associated medical complications [2] and a significant reduction in health-related quality of life [3]. The partial ego-syntonic nature of the disease and a commonly found ambivalence towards change contribute to the maintenance of the disorder and difficulties in its treatment [4, 5]. Only about half of the patients achieve full recovery [6], a percentage that probably has not changed much over the last decades [7]. Thus, the prognosis of AN is still relatively poor. Even though the empirical research on effective treatment for AN is growing, there is still a need to further advance and optimize common therapeutic approaches [5, 8], especially by taking into account the specific mechanisms underlying the disease, which include biological, cognitive as well as social-emotional factors [9, 10]. In this context, increasing attention has recently been placed on the mentalization model and how it can be employed for both, the conceptualization and the treatment of AN and other eating disorders (ED) (e.g [11–13]).

The construct of mentalizing refers to the ability to be aware of mental states such as thoughts, feelings, wishes, and intentions in oneself and others, and to perceive and interpret these mental states in relation to how they underly and, thus, explain human actions and behaviors [14]. Mentalizing is thought to play a pivotal role not only in the regulation and organization of the self, but also in the formation of interpersonal relationships and greater social arrangements [15, 16]. Due to its broad overlap with various related constructs and abilities, such as empathy, Theory of Mind (ToM), alexithymia, and mindfulness, mentalizing is often viewed as an umbrella concept [15]. Mentalizing is a complex and multi-facetted capacity. As evidenced by neuroscientific research, the following four distinct dimensions of mentalizing can be differentiated [15, 17]: (1) *Automatic* vs. *controlled* mentalizing, (2) *cognitive* vs. *affective* mentalizing, (3) mentalizing *oneself* vs. mentalizing *others* and (4) mentalizing with focus on *external* indicators vs. mentalizing based directly on (assumed) *internal* processes. According to the mentalizing model, different mental disorders show differences in the set of imbalances between these dimensions or a certain mentalizing profile that is distinctive for the respective disorder [14, 17]. Furthermore, mentalizing is described as a dynamic, interactive capacity that is both context and relationship dependent and typically increasingly difficult to access in situations with heightened, often attachment-related arousal [14, 15]. In situations of high emotional arousal, mentalizing becomes disrupted and individuals tend to backslide into *prementalizing* modes of subjective experience that developmentally precede the full, mature capacity for mentalizing [14, 17]. These prementalizing modes include the *psychic equivalence mode* (inner, mental experiences and external reality are equalized), the *teleological mode* (mental states are only recognized if they are accompanied by an exterior, observable indicator) and the *pretend mode* (mental states and outer reality are disconnected) [15, 17]. When it resurfaces in adulthood, the pretend mode often involves pseudomentalizing, which describes a state, in which individuals present elaborate, apparently reflective narratives on mental states that are in fact not connected to the persons’ subjective reality and therefore ultimately remain insignificant to them [14].

The ability to mentalize is operationalized as Reflective Functioning (RF) and can be measured with the Reflective Functioning Scale (RF-Scale [18]). While its application to transcripts of the Adult Attachment Interview (AAI [19]) is considered the gold standard measure for the assessment of mentalizing, the RF-Scale (or slightly adapted versions of it) can also be coded based on other semi-structured interviews such as the Brief Reflective Functioning Interview (BRFI [20]) or it can be used for the RF-rating in therapy sessions [21].

Besides the interview based coding systems, a range of other methods for an assessment of RF is available, including self-report measures, experimental tasks or performance-based measures [22].

Within the mentalizing model, eating disorders (EDs) such as AN and bulimia nervosa (BN) are conceptualized as “self-disorders” [13]: In light of an inadequately integrated psychic reality, the body functions as medium for the expression of unprocessed states of mind and might thereby acquire an excessive importance for the maintenance of an overall weak sense of self, ultimately leading to a wide range of typical ED symptoms [11, 16]. It is assumed, that the impaired mentalizing abilities in patients with an ED come along with a vast occurrence of prementalizing modes [11, 13]. The way in which these these prementalizing modes (i.e. the mode of psychic euquivalence, the teological mode and the pretend mode) are related to ED symptomatology can be illustrated by the following clinical examples:

First example: To a patient operating in the mode of psychic equivalence, the *subjective* impression of “feeling fat” might equate with *objectively* “being fat”. The possibility that the inner, subjective reality might not correspond to the outer, objective reality of being underweight, which can sometimes even be life-threatening, is ignored [23]. Second example: When a person diagnosed with an ED uses the rigid restriction of food intake as a means of controlling overwhelming emotions, or when she attempts to improve her social acceptance and self-esteem by changing her own bodily appearance through weight loss (visible as a lower number on the scale), these behaviours reflect a teological stance. Something concrete and visible is needed to express and understand complex inner states [24]. Third example: A patient operating in pretend mode may engage in detailed and seemingly reflective conversations about her inner states related to her ED during therapy sessions. However, this seemingly newly gained awareness remains detached from reality and therefore does not lead to a change of behaviour or a remission of ED symptoms [11, 24].

In line with this, previous research suggests, that the capacity to mentalize is impaired in people diagnosed with AN [25]. Compared to healthy individuals, patients with AN seem to be characterized by lower RF-levels in interview-based assessments of their overall mentalizing abilities [26–28] and higher levels of alexithymia [29], which refers to difficulties in identifying and describing one’s own emotions. Mesaures of alexithymia can be regarded as proxy measures for mentalizing in the self-dimension [15]. However, the findings are less consistent regarding the ability to mentalize with respect to others which e.g. can be captured by assessing the related construct of ToM [22]: While two older meta-analysis found deficits in ToM or the understanding of mental states in others, respectively, in patients with AN compared to healthy individuals [29, 30], some more recent reviews are more skeptical regarding possible impairments in ToM or in “mentalizing others” in patients with AN [25, 31]. Additionally, there is some evidence indicating that patients with AN might not be homogenous regarding deficiencies in both overall mentalizing and its dimensions, but that different subgroups with varying mentalizing profiles have to be differentiated [32, 33]. Harrison et al. [34], for example, found that impairments in the ability to recognize emotions in others were more severe in patients with restricting AN than in patients with binge-purging AN, suggesting that the subtype of the illness might play a role when discussing mentalizing profiles in AN. Another study by Rommel et al. [35] points in the same direction: Only patients with the restricting subtype of AN demonstrated difficulties in the emotional awareness of others, while patients with binge-purging behavior (binge-purging AN and bulimia nervosa) did not differ significantly from HCs.

Furthermore, there is some evidence that greater impairments in the capacity to mentalize are at least to some extent associated with a greater severity of ED symptoms (e.g. the desire to be thin, an intense fear of gaining weight or the constant preoccupation with food and dieting) and/or ED related psychological traits (e.g. emotional and interpersonal problems or a low self-esteem) [26, 27]. However, findings on the association of mentalizing and ED symptomatology are still inconsistent as some studies found no correlation between these variables (e.g [36, 37]). Moreover, the relation between mentalizing abilities and other aspects of psychopathology, such as alterations in body experience or impairments in personality functioning which shares a broad conceptual and empirical overlap with the construct of mentalization [38–41], has rarely been studied in this patient group. Additionally, given that AN is frequently associated with a wide range of psychiatric comorbidities [42] including affective and anxiety disorders which in turn have been linked to impairments in the ability to mentalize [43–45], a connection between low RF-levels and comorbid depressive and anxiety symptoms, respectively, might be worth examining.

Recently, in both research and clinical practice there has been a broad use of self-report measures such as the *Reflective Functioning Questionnaire* [46] and the *Mentalization Questionnaire* [47] for the assessment of the overall capacity to mentalize. However, even though those measures are well validated, evidence of their relation to an observer-rating on the RF-Scale is still scarce. At the same time, in light of the frequent conceptualization of AN and other EDs as “Self-Disorders” [13], combining an expert-rating with the patients’ own view might be of particular interest for the assessment of mentalizing and its relation to psychopathology in this particular group of patients.

### Study aims and hypotheses

The first aim of the current study was to compare the mentalizing abilities of patients with AN to a HC group using both an observer-rated as well as a self-report measure to capture RF. We hypothesized that participants with AN will show lower RF levels in an expert rating as well as in a self-report measure compared to HCs.

The study’s second aim was to examine the association between the patients’ mentalizing abilities and various aspects of ED specific psychopathology (e.g. eating, weight and shape concerns, drive for thinness) and general psychopathology including, disturbances in body experience, depressive symptoms and anxiety as well as overall personality functioning. We postulated that lower levels in RF will be associated with more ED specific symptomatology, with greater disturbances in the aspects of general psychopathology mentioned above as well as with a lower BMI.

Finally, it was aimed to explore possible differences in mentalizing capacities between AN-subtypes.

The study was part of a larger study on mentalizing and body experience in patients with an ED.

## Methods

### Study design

The study was designed as a cross-sectional comparative study, using an interview-based assessment and self-report questionnaires.

A sample of adult female patients with AN was compared with a matched non-clinical control group of healthy adult females. For a multi-method RF measurement, an expert rating and a questionnaire (self-report) were combined. In terms of psychopathology, the following aspects were examined in the AN group (self-report): ED psychopathology including BMI, personality functioning, body experience, depressive symptoms and anxiety. The participants filled in a set of questionnaires and afterwards took part in a video-recorded interview.

The study was approved by the local ethics committee of the University Hospital Freiburg (vote 22-1096; 22-1096_1).

### Participants

*The clinical group* included 30 female, adult patients with AN, recruited at the Department of Psychosomatic Medicine and Psychotherapy, Freiburg University Hospital, Germany and at private psychotherapy practices over a period of 2 years. All patients met the ICD-10 [48] criteria for either AN (F50.0) or atypical AN (F50.1).

Inclusion criteria were female gender, an age between 18 and 60 years and written consent to participate in the study. Exclusion criteria were a current or lifetime diagnosis of psychotic disorder, bipolar disorder, substance abuse disorder, intellectual disability or organic brain disease as well as an insufficient understanding of the German language. Diagnoses (including the AN diagnosis) were ascertained by experienced clinicians according to ICD-10 after a comprehensive clinical interview in the outpatient clinic.

The *control group* consisted of 30 healthy, female adults that were recruited among university students and employees of the University Hospital Freiburg. They were required to have no lifetime history of any psychiatric illness (participants were asked about past psychiatric diagnoses as part of the screening procedure) and to show no indication of a current ED or another current mental disorder in two screening questionnaires (SEED, PHQ, see below).

Since both study groups were required to match in rough age (+/− 3 years) and educational level (highest school education), the HCs were recruited gradually over time subsequently to the patients: At several points during the study, requests for participation in the study were published. In chronological order of their application, interested healthy subjects were screened for a potential “match” with an already interviewed patient and, if appropriate, were invited for further screening and information.

Both the patients and the HCs received 30 Euros for their participation in the study.

### Measures

#### The Reflective Functioning Scale (RF-Scale)

The RF-Scale [18] was developed for the assessment of RF based on transcripts of the AAI [19]. The scale ranges from − 1 to 9, with only the odd numbers representing defined categories, while the even numbers serve as intermediate stages allowing for a more refined evaluation [49]. A rating of −1 (“negative RF”) refers to bizarre, inappropriate or unintegrated mental state attributions or to a hostile, rejecting stance towards RF, while a score of 9 (“exceptional RF”) corresponds to an extraordinarily sophisticated, original and consistent understanding of mental states and the way in which they underlie behavior. A score of 5 (“ordinary RF”), however, is supposed to be the most common rating in a healthy population and requires clear examples of mentalizing, even if RF remains relatively simple. In order to facilitate the rating, the *Reflective Functioning Manual* [18] provides the following qualitative markers as indicators for the presence of moderate to high RF (scores of ≥ 4): (1) Recognizing the characteristics of mental states, including their opaqueness and their susceptibility to disguise, (2) making an effort to find plausible links between behaviors and underlying mental states, (3) being aware of developmental aspects of mental states and (4) demonstrating an understanding of mental states in relation to the interviewer.

In the course of the scoring procedure, the questions of the interview transcript are first rated individually on the RF-Scale. In a second step, the RF-ratings of the individual questions are aggregated into a global score with the main focus being on the results from the demand questions (i.e. questions that explicitly provoke the demonstration of RF rather than just allow it). However, the global score does not simply represent the arithmetic mean of the single ratings, but rather it’s obtained by regarding the interview as a whole and individually weighting each passage [18, 50].

#### The Brief Reflective Functioning Interview (BRFI)

The BRFI [20] is a short, semi-structured interview designed to capture mentalizing on the RF-Scale. It was developed on the basis of the AAI [19] with the intention to reduce administration, transcription and coding times and is therefore thought to provide a time- and costeffective alternative to the AAI for the interview-based assessment of RF [51]. The english version of the BRFI comprises a total of eleven, the german version a total of ten questions, the first eight of which refer to one freely chosen parent. Participants are asked to reflect on the personality of this parent, on their relationship with him or her and on the ways in which this parent has influenced their lives. The final two questions require the interviewees to deliberate on another person who is currently important to them in order to allow an assessment of their mentalizing in the context of a non-parental relationship [52].

RF is coded on the RF-Scale in accordance with the general principles outlined in the *Reflective Functioning Manual* [18] that were described above (in the following named “RF-BRFI”). However, since every question of the BRFI is designed to explicitly prompt RF, all of them are rated as demand questions (in contrast to the differentiation between *demand* and *permit* questions in the AAI) and must therefore be considered in the formation of the global rating [51].

In several studies, the RF-scores obtained using the BRFI show a strong correlation with those obtained using the AAI (*r* = 0.71 to 0.88), indicating the validity of the measure [51, 52]. Both the English and the German version of the BRFI have shown an excellent internal consistency (Cronbach’s α = 0.92 to 0.97) and a good interrater reliability for the total score (ICC = 0.79 to 0.85) [51, 52].

The mean duration of the BRFI in this study was 15 min 48 s (SD = 6 min 18 s) for the patients and 20 min 9 s (SD = 11 min 10 s) for the control group (t(58) = − 1.86; *p* = 0.07).

#### The Mentalization Questionnaire (MZQ)

The MZQ [47] is a self-rated instrument originally designed for the assessment of mentalization in patients with mental disorders. It consists of 15-Items that are answered on a five-point Likert scale ranging from 1 (“don’t agree at all”) to 5 (“agree completely”) and that can be assigned to the following four subscales: “refusing self-reflection”, “emotional awareness”, “psychic equivalence mode” and “regulation of affect”. However, for the purpose of this study only the total score of the MZQ representing the arithmetic mean of all its items was used for further analyses. It is important to note that, in contrast to the RF-Scale, higher scores in the MZQ indicate greater impairment in the capacity to mentalize.

The reliability of the MZQ-total score is good, with an internal consistency of α = 0.81 and a retest-reliability of *r* = 0.76 in the original evaluation of the questionnaire in a clinical population [47] and similar results for the assessment in a non-clinical population [39]. Convincing evidence for the convergent and divergent validity of the measure was found in several studies using both clinical and community samples [39, 47].

#### Eating Disorder Inventory, second version (EDI-2)

The EDI-2 [53, 54] is a self-report questionnaire that measures behavioral and attitudinal components of eating disorder psychopathology. The original version of the questionnaire consists of 64 items that are answered on a six-point Likert scale and generate the following 8 subscales addressing both core eating disorder symptomatology and associated psychological traits: “drive for thinness”, “bulimia”, “body dissatisfaction”, “ineffectiveness”, “perfectionism”, “interpersonal distrust”, “interceptive awareness” and “maturity fears”. With regard to the items’ polarity, sum scores are calculated for each subscale with higher scores indicating a greater severity of ED psychopathology. The EDI-2 has repeatedly demonstrated good validity and reliability. For patients with an ED, all EDI-2 subscales showed excellent internal consistencies (α = 0.82 to 0.90 [53]).

#### Eating Disorder Examination-Questionnaire (EDE-Q)

The EDE-Q [55] is a self-report measure for the assessment of eating disorder psychopathology and behaviors in the past 28 days. It contains 22 items that address core attitudinal aspects of eating disorder psychopathology and are scored on a seven-point rating scale ranging from 0 (“no days”) to 6 (“every day”). Those 22 items constitute four subscales (“restraint”, “eating concern”, “weight concern” and “shape concern”) which are computed by calculating the average of the contributing items. In addition, a total score representing the mean of all 22 items can be obtained. For both the subscales and the global score, higher values indicate a greater severity of symptomatology.

Furthermore, the EDE-Q contains 6 additional items to assess eating disorder behaviors (e.g. self-induced vomiting, binge eating, misuse of laxatives) in terms of their frequency within the past 28 days. Those items do not contribute to any of the subscales or the global score.

The German version of the EDE-Q [56] showed a good internal consistency (α = 0.85 to 0.97), significant test-retest correlations (*r* = 0.67 to 0.88) and strong indications for convergent und discriminant validity for both the subscales and the total score [57].

#### Short Evaluation of Eating Disorders (SEED)

The SEED [58] is a self-rated measure developed for a fast assessment of core eating disorder symptomatology. It comprises six items that allow the calculation of a total severity index for AN and BN symptoms, respectively, ranging from 0 (“no symptoms”) to 3 (“extreme symptoms”). The SEED was validated for both a clinical and a non-clinical population [58].

In this study, the SEED was used to screen for possible current ED symptoms in the HC group. HCs surpassing a total severity score of 1 for either AN or BN were excluded from the study.

#### Operationalized Psychodynamic Diagnosis-Structure Questionnaire Short Form (OPD-SQS)

The OPD-SQS [59] is a self-report measure for the assessment of personality functioning based on the Level of Structural Integration Axis by the OPD. The questionnaire comprises 12 Items to be answered on a five-point scale ranging from 0 (“fully disagree”) to 4 (“fully agree”). In the end, a total sum score (0 to 48) is calculated with higher values indicating a more severe personality dysfunction.

The OPD-SFK has shown good reliability and validity for both clinical and non-clinical samples [59, 60]. The internal consistency for the total scale was α = 0.88 in the original sample [59].

#### Dresden Body Image Inventory-35 (DKB-35)

The DKB-35 [61] is a self-report measure to assess five dimensions of body experience. We prefer the term “body experience” over the term “body image”, for the dimensions of the DKB-35 cover a broader concept of how the body is perceived and experienced subjectively rather than just “imaged”. The dimensions covered in the DKB-35 include “vitality”, “self-acceptance”, “sexual fulfillment”, “self-aggrandisement” and “physical closeness”. The 35 items of the DKB-35 are rated on a five-point Likert scale. Subscale-scores are computed by calculating the average score of all contributing items, respectively. With regard to the items’ polarity, higher scores indicate a more positive body experience. Reliability and validity of the DKB-35 was confirmed for both clinical and non-clinical samples [62, 63].

#### Patient Health Questionnaire (PHQ) and Generalized Anxiety Disorder Scale-7 (GAD-7)

Parts of the PHQ [64, 65] were applied for an assessment of general psychopathology: the PHQ-9 [66] was used to evaluate symptoms of depression, the PHQ-15 [67] was administered to capture somatic symptoms and the GAD-7 [68] was applied to assess symptoms of anxiety. The items of the PHQ-9 and the GAD-7 are answered on a four-point scale, ranging from 0 (“not at all”) to 3 (“nearly every day”), while the 15 somatic symptoms of the PHQ-15 are scored from 0 (“not bothered at all”) to 2 (“bothered a lot”). For both the PHQ-15 and the GAD-7, scores of 5, 10, 15 represent cutoff points for mild, moderate and severe symptom severity, respectively. For the PHQ-9, scores of 5, 10, 15, 20 indicate the presence of mild, moderate, moderately severe and severe depressive symptoms, respectively. The PHQ-9, the PHQ-15 and the GAD-7 were all validated and demonstrated an excellent internal consistency with a Cronbach’s α of 0.89, 0.80 and 0.92, respectively [66–68].

For our sample, the Cronbach’s α ranges between 0.76 and 0.89, with the exeption of the EDI-2 subscale “interpersonal distrust” (0.64) and the DKB-35 subscale “self-aggrandisement” (0.66) (see Tables 1 and 2). Given the small sample size this indicates acceptable to very good reliability.

### Procedure

After a comprehensive explanation of the study’s objectives and methodology, as well as the procedures for ensuring data protection, all participants provided their written informed consent to participate in the study.

The study measurements were then carried out by two students/research assistents, who had previously been trained by experienced clinicians.

First, the participants were asked to complete a series of questionnaires (AN group: MZQ, EDI-2, EDE-Q, OPD-SQS, PHQ-9, PHQ-15, GAD7, DKB-35; HC group: MZQ, SEED, PHQ-9, PHQ-15, GAD-7).

Subsequently, the BRFI was administered in random order along with an interview about the participants’ body experience which was used for another study.

The interviews were recorded using two small cameras, one focused on the interviewer and the other one on the interviewee, and then transcribed using the audio tracks from the videos. The BRFI transcripts were then coded on the RF-Scale by two of the authors (IL, AZ) who are trained and reliable raters of the RF-scale. Difficult passages with deviations in the ratings were discussed with the other rater, and consensus was reached. Both raters were blind to the interviewees’ group allocation and to the results of the self-report measures.

The reliability of IL and AZ for ratings of BRFI-transcripts on the RF-scale was confirmed prior to this study by a comparison of their own ratings with a gold standard. This gold standard was created by LH and a collegue, two certified RF raters. For this purpose, 10 BRFIs were rated and a consensus was reached. The interrater-correlation between IL and the gold standard was 0.82 (*p* < 0.001) and between AZ and the gold-standard 0.90 (*p* < 0.001), respectively.

### Statistical analysis

Means, standard deviations and frequencies were calculated for the description of the sample. For the EDI-2, a T-standardization based on the representative sample of Kordy et al. [69] was performed to provide easily interpretable scores (A T-value of 50 is equal to the mean of the representative sample, +/− 10 T-points equal +/1 one SD).

Between-group differences in sociodemographic characteristics were analyzed using χ^2^-tests. In order to compare group means for age, BMI and mentalizing variables, we employed one- and two-sided t-Tests for independent samples after testing for variance homogeneity with Levene’s tests. Cohen’s d (d) was calculated for the between-group comparison of mentalizing abilities. Effect sizes of $\left| {\text{d}} \right|$ = 0.2, $\left| {\text{d}} \right|$ = 0.5 and $\left| {\text{d}} \right|$ = 0.8 were considere small, medium and large effect sizes. Pearson correlation coefficients (r) were used to examine and quantify the association between numeric variables. Effect sizes of $\left| {\text{r}} \right|$ = 0.10, $\left| {\text{r}} \right|$ = 0.30 and $\left| {\text{r}} \right|$ = 0.50 were considered small, medium and large in magnitude [70].

Since this study was partially exploratory and hypothesis generating, we did not adjust the alpha level (α = 0.05) for multiple testing. Instead, we considered effect sizes and the explained variance in the interpretation of the statistical results. Analyses were conducted in SAS-JMP, version 13.2.1.

## Results

### Sample

Table 1 shows sociodemographic and clinical characteristics of the two study groups. There was no significant difference in age between the AN group [Range: 18–57 years) and the HC group (range: 20–57 years; t(58) = 0.46; *p* = 0.644]. School education was distributed equally. There were no significant between-group differences in school education (perfect match), professional education (χ^2^ = 6.9; df = 3; *p* = 0.076) or marital status (χ^2^ = 0.41; df = 2 *p* = 0.812). As expected, the mean BMI was significantly lower in the AN group (Range: 13.4–23.8 kg/m^2^) than in the HC group (Range: 18.7–33.9 kg/m^2^; t(58) = 7.33; *p* < 0.0001).

**Table 1:** General characteristics of participants with AN and HCs

Sociodemographic characteristics	AN (*N* = 30)		HC (*N* = 30)		
	M (SD)	N (%)	M (SD)	N (%)	
Age (years)	26.2 (10.1)		27.4 (9.9)		
School education					
9 years		1 (3.3)		1 (3.3)	
10 years		6 (20)		6 (20)	
12 years*		23 (76.7)		23 (76.7)	
Professional education					
Ongoing		16 (53.3)		11 (36.7)	
Apprenticeship		7 (23.3)		9 (0.3)	
University degree		5 (16.7)		10 (33.3)	
Without degree		2 (6.7)		0 (0)	
Marital status					
Single		22 (73.3)		22 (73.3)	
Married/with partner		6 (20)		7 (23.3)	
Separated/divorced/widowed		2 (6.7)		1 (3.3)	

Clinical characteristics	AN (*N* = 30)		HC (*N* = 30)		Cronbach’s α
	M (SD)	N (%)	M (SD)	N (%)	
BMI (kg/m^2^)	17.1 (2.2)		22.2 (3.1)		
SEED					
SEED—AN total severity index			0.28 (0.19)		
SEED—BN total severity index			0.09 (0.20)		
General psychopathology					
PHQ-9—Depressive symptoms**	10.83 (7.84)		0.27 (0.87)		0.89
PHQ-15—Somatic symptoms**	12.76 (5.40)		4.43 (2.84)		0.79
GAD-7—Anxiety**	11.34 (5.04)		2.10 (1.79)		0.87

*AN* Anorexia nervosa; *HC* Healthy control; *N* sample size; *M* mean; *SD* standard deviation; *BMI* Body mass index; *BN* Bulimia nervosa; *SEED* Short evaluation of eating disorders; *PHQ* Patient Health Questionnaire; *GAD-7* Generalized Anxiety Disorder 7

^*^3 patients who were in their 12th school year and about to take their final exams were also allocated in this category; ^**^ *N* = 29

As expected, the AN group differed significantly from the HC group regarding depressive symptoms (t(28.7) = − 7.21; *p* < 0.001), somatic symptoms (t(42.1) = − 7.38; *p* < 0.001) and anxiety (t(34.7) = − 9.32; *p* < 0.0001).

Clinical history and psychopathology of participants with AN are shown in Table 2. The participants with AN were diagnosed with either AN restricting type (*n* = 14), AN binge-purge type 8 (*n* = 7) or atypical AN (*n* = 9). Out of the nine participants with atypical AN, six showed a restrictive symptomatology and three binge eating and/or purging behavior. Comorbid diagnoses in the AN group included affective disorders (*n* = 16; 53.3%), posttraumatic stress disorder (*n* = 6; 20%) and anxiety disorders (*n* = 2; 6.7%).

**Table 2:** Clinical history and psychopathology of participants with AN

Clinical history	Patients with AN (*N* = 30)		
	M (SD)	N (%)	
Duration of illness* (years)	6.4 (5.9)		
Current treatment setting			
Outpatient		16 (53.3)	
Day hospital		4 (13.3)	
Hospital		10 (33.3)	
Previous treatments			
Total number	2 (2.7)		
None		10 (33.3)	
≥ 1		20 (66.7)	

Psychopathology	Patients with AN (*N* = 30)		Cronbach’s α
	M (SD)	N (%)	
Eating disorder symptomatology			
EDE-Q			
EDE-Q—Total score	3.66 (1.23)		0.82
EDE-Q—Restraint	3.34 (1.50)		0.76
EDE-Q—Eating concern	3.25 (1.30)		0.72
EDE-Q—Weight concern	3.65 (1.59)		0.83
EDE-Q—Shape concern	4.40 (1.34)		0.88
EDI			
EDI—Drive for thinness	80.94 (21.65)		0.79
EDI—Bulimia	53.83 (13.18)		0.77
EDI—Body dissatisfaction**	68.25 (11.11)		0.78
EDI—Ineffectiveness	76.48 (17.56)		0.78
EDI—Perfectionism	62.11 (13.49)		0.76
EDI—Interpersonal distrust	53.33 (12.53)		0.64
EDI—Interoceptive awareness	73.69 (18.00)		0.77
EDI—Maturity fears	59.09 (16.45)		0.84
Personality functioning			
OPD-SQS	27.30 (9.87)		0.88
Body experience			
DKB-35			
DKB—Vitality	2.64 (0.70)		0.83
DKB—Self-acceptance	1.96 (0.63)		0.80
DKB—Sexual fulfillment	1.93 (1.01)		0.93
DKB—Self-aggrandisement	1.98 (0.57)		0.66
DKB—Physical closeness	3.09 (0.81)		0.84

*AN* Anorexia nervosa; *N* sample size; *M* mean; *SD* standard deviation; *EDE-Q* Eating Disorder Examination Questionnaire; *EDI* Eating Disorder Inventory; *OPD-SQS* Operationalized Psychodynamic Diagnosis-Structure Questionnaire, Short Version; *DKB-35* Dresden Body Image Inventory-35

^*^Duration of illness was calculated from the (reported) onset of significant AN symptoms. ^**^ *N* = 29. For the EDI-Scores, a T-standardization was performed based on the representative sample of Kordy et al. [69]

### Between-group differences in mentalizing

**Fig. 1:**
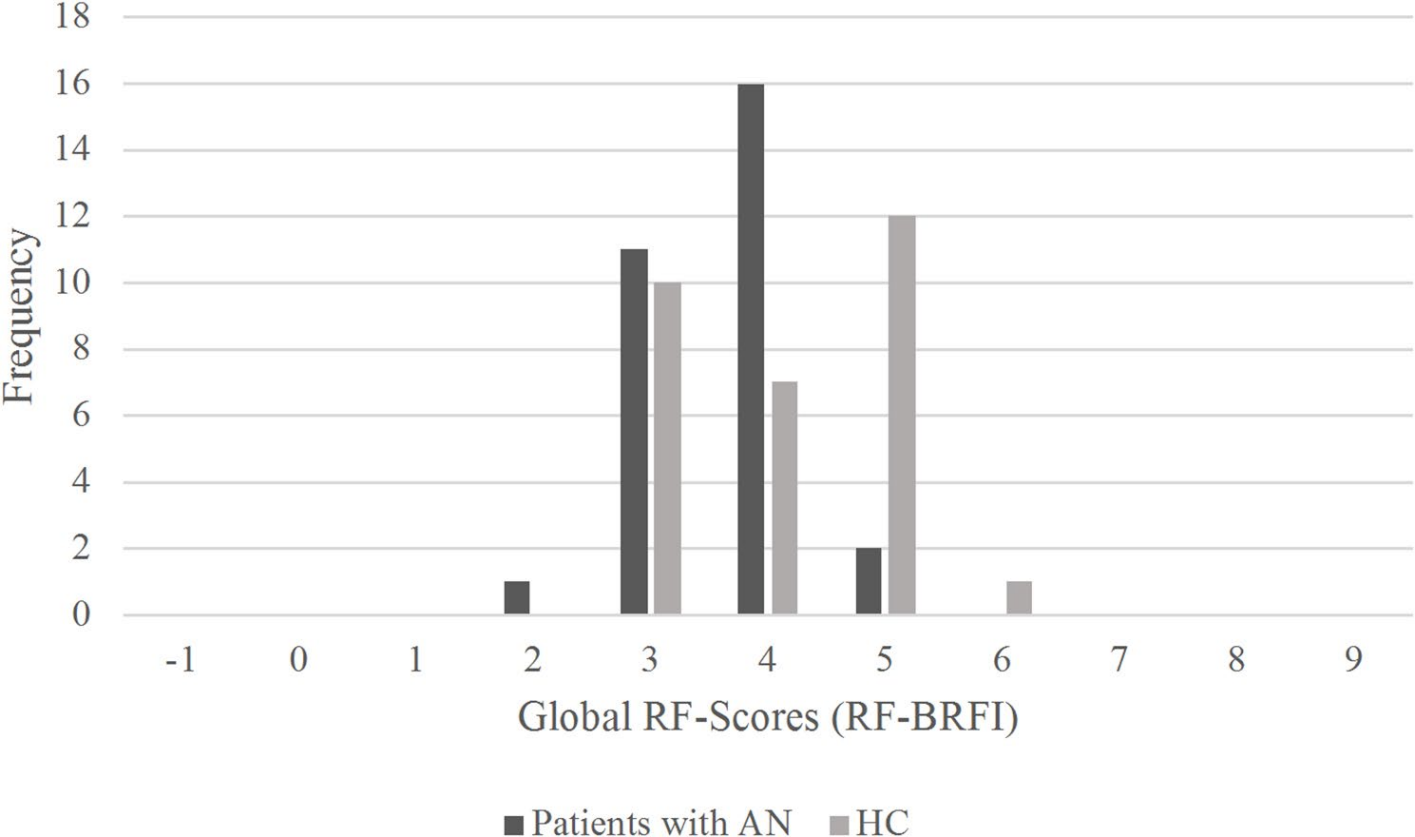
Distribution of global RF-Scores on the BRFI. Frequency of expert-rated global RF-Scores are indicated in dark grey for Patients with AN and in light grey for HCs. The most common rating for patients with AN was RF = 4 while for HCs it was RF = 5. *RF* Reflective Functioning; *BRFI* Brief Reflective Functioning Interview; *AN* Anorexia nervosa; *HC* Healthy control

The distribution of the global ratings on the RF-Scale for both groups is depicted in Fig. 1.

For a comparison of group means of the RF-BRFI and of the self-reported mentalizing in the MZQ see Table 3. In line with the first hypothesis, results showed, that the mean RF-BRFI value was significantly lower for the AN group than for the HC group. Furthermore, and also in accordance with our first hypothesis, there was a significant difference between the groups in terms of the MZQ total score. The AN group showed significantly worse self-reported mentalizing capacities (i.e. higher scores in the MZQ) than the HC group.

**Table 3:** Between-group differences in mentalizing

	HC M (SD) (*N* = 30)	AN M (SD) (*N* = 30)	t	df	*p*	d
RF-BRFI	4.13 (0.94)	3.63 (0.67)	2.38	52.5	0.011	0.61
MZQ-total score*	1.73 (0.45)	3.24 (0.71)	− 9.38	49.1	< 0.0001	− 1.80

*HC* healthy control; *AN* anorexia nervosa; *M* mean; *SD* standard deviation; *df* degrees of freedom; *d* Effect size; *N* sample size; *RF* Reflective Functioning; *BRFI* Brief Reflective Functioning Interview; *MZQ* Mentalization Questionnaire

^*^Missing data for 8 HCs: N(HC) = 22; Expecting mentalizing to be impaired in the patient sample, t-Tests were performed one-sided

### Association of mentalizing with ED symptoms and aspects of general psychopathology

For correlations between ED symptomatology (EDE-Q, EDI-2, BMI), aspects of general psychopathology (personality functioning, depression, anxiety, somatic symptoms) as well as body experience and the two mentalizing measures (RF-BRFI and MZQ total score) in the AN group see Table 4.

**Table 4:** Correlation between mentalizing and dimensions of psychopathology in AN participants

	RF-BRFI *r*	MZQ-total score *r*
Eating disorder symptomatology		
EDE-Q		
EDE-Q—Total score	− 0.31	0.40*
EDE-Q—Restraint	− 0.10	0.01
EDE-Q—Eating concern	− 0.26	0.51**
EDE-Q—Weight concern	− 0.39 *	0.45*
EDE-Q—Shape concern	− 0.33	0.41*
EDI-2		
EDI-2—Drive for thinness	− 0.34	0.31
EDI-2—Bulimia	− 0.14	0.34
EDI-2—Body dissatisfaction^1^	− 0.29	0.35
EDI-2—Ineffektiviness	0.05	0.63***
EDI-2—Perfektionism	0.34	0.35
EDI-2—Interpersonal distrust	0.02	0.62***
EDI-2—Interceptive awareness	− 0.15	0.80***
EDI-2—Maturity fears	− 0.13	0.37*
BMI	0.02	0.00
Personality functioning		
OPD-SQS total score	0.01	0.79***
General psychopathology		
PHQ-9^1^	− 0.11	0.30
PHQ-15^1^	0.00	0.30
GAD-7^1^	− 0.25	0.65***
Body experience		
DKB-35—Vitality	− 0.28	− 0.16
DKB-35—Self-acceptance	0.20	− 0.41*
DKB-35—Sexual fulfillment	0.17	− 0.58***
DKB-35—Self-aggrandisement	0.10	− 0.44*
DKB-35—Physical closeness	0.21	− 0.54**

Sample size (N) was *N* = 30 with the exceptions of the instances marked with ‘^1^’ where sample size was *N* = 29

*RF* Reflective Functioning; *BRFI* Brief Reflective Funcitoning Interview; *MZQ* Mentalization Questionnaire; *EDE-Q* Eating Disorder Examination-Questionnaire; *EDI-2* Eating Disorder Inventory-2; *BMI* Body-Mass-Index ; *OPD-SQS* Operationalized Psychodynamic Diagnosis-Structure Questionnaire, Short Version; *PHQ* Patient Health Questionnaire; *DKB-35* Dresden Body Image Inventory-35

^*^p < 0.05; ^**^p < 0.01; ^***^p < 0.001

Regarding the RF-BRFI, a significant negative correlation was found only for the subscale “weight concerns” of the EDE-Q.

Regarding the association of the MZQ total score and the EDE-Q-variables, significant positive correlations with moderate to large effect sizes were found for the EDE-Q total score as well as for subscales “eating concerns”, “weight concerns” and “shape concerns”. Among the EDI-subscales, the subscales “ineffectiveness”, “interpersonal distrust”, “interceptive awareness” and “maturity fears” were significantly correlated with the MZQ total score. No significant association was found between both measures of mentalizing and the BMI.

Personality functioning as measured with the OPD-SQS was significantly positively and with a large effect size correlated with the MZQ total score, indicating that a lower level of self-reported mentalizing was associated with more severe impairment in personality functioning. However, no significant correlation was found between the OPD-SQS and the RF-BRFI.

Neither expert-rated nor self-reported mentalizing was significantly correlated with symptoms of depression as measured with the PHQ-9 or with general somatic symptoms (PHQ-15). However, the level of anxiety as measured with the GAD-7 correlated significantly and with a large effect size with the MZQ total score, but not with the RF-BRFI.

Furthermore, there was no significant correlation between expert-rated mentalizing (RF-BRFI) and either one of the DKB-35-subscales for the assessment of body experience. In contrast, self-reported mentalizing (MZQ total score) was negatively and with moderate to large effect sizes associated with different aspects of body experience as assessed with the DKB-35.

### Mentalizing in AN-subgroups

While there were no significant differences between the restrictive and the binge-purging subgroup in the RF-BRFI values (t(28) = 0.38; *p* = 0.707), patients with restrictive behavior (*N* = 20; M = 3.03; SD = 0.76) showed significantly better self-reported mentalizing (i.e. lower scores in the MZQ total score) than patients with binge-purge behavior (*N* = 10; M = 3.67; SD = 0.31; t(27.3) = 3.28; *p* = 0.003).

### Additional analysis: relationship between RF-BRFI and MZQ

There was a significant difference in RF between the AN and the HC group for both the MZQ total score and the BRFI. However, while there was a medium effect size of the between group-difference regarding the BRFI-related measure, the effect size of the between group-difference regarding the MZQ was large. In addition, the correlations between mentalizing and dimensions of psychopathology in AN participants varied for the different methods of RF assessment. We therefore decided to perform an additional analysis of the data that was not originally mentioned in our study proposal and examined the relationship between the RF-BRFI and the MZQ total score using Pearson correlation coefficients.

**Fig. 2:**
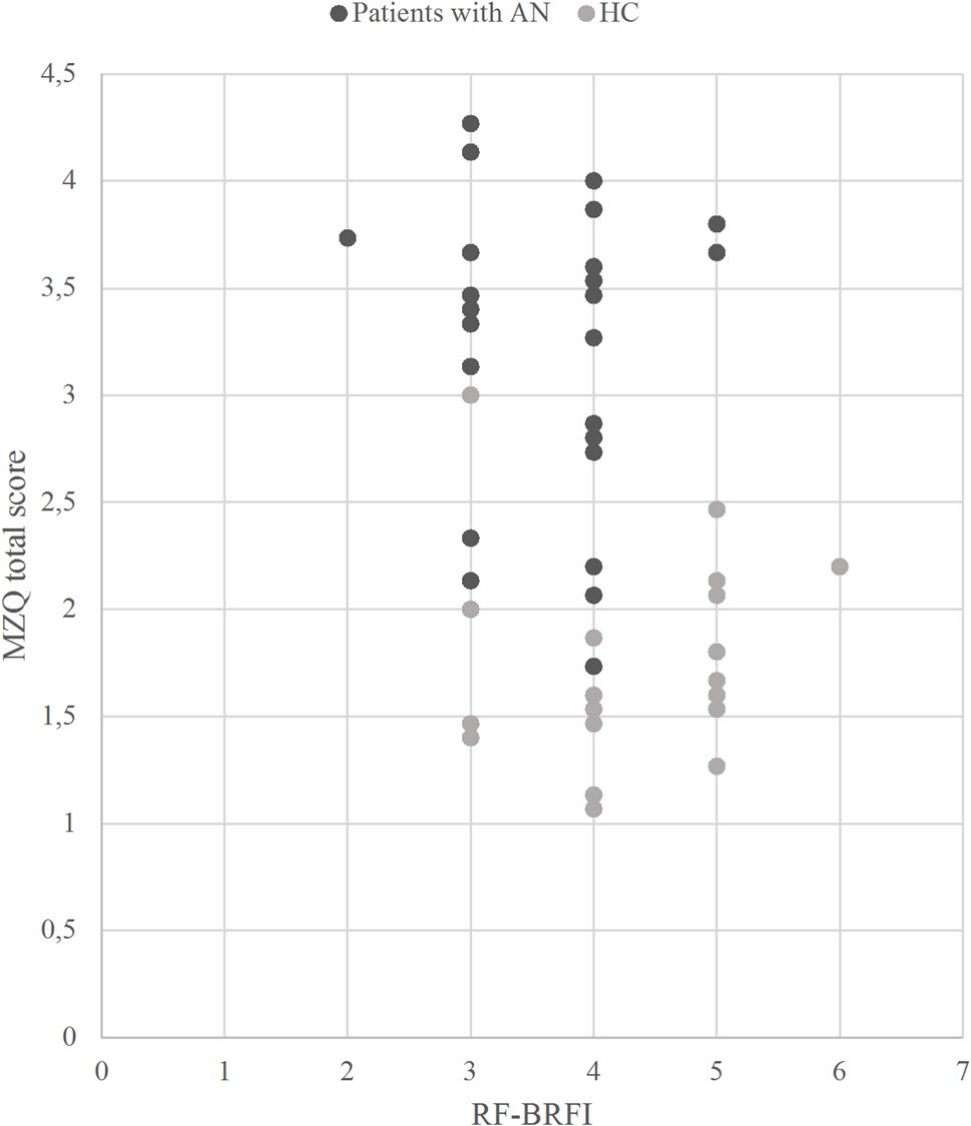
Association between the RF-BRFI and the MZQ total score. Expert-rated global RF-Scores (RF-BRFI) and correspondant scores of self-reported mentalizing (MZQ total score) are indicated in dark grey for participants with AN and in light grey for HCs. While for the whole sample, a significant negative correlation with moderate effect size was found between the RF-BRFI and the MZQ total score indicating that higher expert-rated RF was associated with better self-reported mentalizing, no significant correlation was found between the two measures of mentalizing within the individual groups. *RF* Reflective Functioning; *BRFI* Brief Reflective Functioning Interview; *MZQ* Mentalization Questionnaire; *AN* Anorexia nervosa; *HC* Healthy control

For the whole sample, a significant negative correlation with moderate effect size was found between the RF-BRFI and the MZQ total score indicating that higher expert-rated RF was associated with better self-reported mentalizing (*r* = − 0.38; *p* = 0.006). However, as visualized in Fig. 2, for both individual groups, no significant correlation was found between the two measures of mentalizing (*r* = 0.07 for the HC group and *r* = − 0.14 for the AN group; both n.s.).

## Discussion

The aim of this study was to examine the level of mentalizing abilities among patients with AN in comparison with matched HCs using both an expert-rated and a self-reported measure for the assessment of RF. Additionally, the study focused on investigating the relation between mentalizing and various aspects of ED-related and general psychopathology.

The matching procedure was fully successful concering age, school education and marital status. Given the same age distribution, the AN sample trended towards more ongoing professional education with less university degrees (*p* = 0.076). We attribute this trend to the interruptions and delays caused by the illness (e.g. inpatient treatments).

In accordance with our hypothesis, RF was significantly lower in the AN group than in the HC group. This is in line with other studies, that also found lower RF levels in patients with AN compared to HCs [26–28]. However, the absolute difference in expert-rated RF (RF-BRFI) between the two groups was relatively small. On the one hand, the mean RF of M = 3.63 in the AN group indicates that the patients did indeed, as expected, demonstrate difficulties recognizing and interpreting mental states. On the other hand, the deficits in their ability to mentalize were not quite as severe as results of other studies suggest [27, 71]. This could be partially attributed to the fact, that this study included a considerable number of outpatients while previous research mostly focused on inpatients with AN (e.g [27, 71]). However, the overall severity of ED-psychopathology in the present sample was still substantial. Another contributing factor could be a methodological one. While in previous studies, the scoring of patients’ mentalizing ability on the RF-Scale was mostly based on transcripts of the AAI, we used the BRFI as an abbreviated version of it. However, the RF-scores obtained by using the AAI and those obtained by using the BRFI have previously been reported to be highly correlated [51, 52]. Therefore, it seems unlikely that the use of the BRFI in the present study would have influenced the results in the direction of higher RF. Another relevant factor could be that the long duration of illness and the high number of previously received treatments which characterizes the patient sample of this study might have impeded and to some extent distorted the assessment of the patients’ RF since it might have led to an increased occurrence of pseudomentalizing. Individuals who pseudomentalize may talk elaborately and at first glance in a mentalizing manner about mental states but their words are severed from their emotional experience and, thus, carry little true meaning [14, 15]. Pseudomentalizing can be difficult to differentiate from genuine mentalizing. Furthermore, it has previously been discussed as a challenge in the psychotherapeutic treatment of patients with AN and other EDs [11, 13] and has also empirically been shown in individuals with AN [72]. It may be possible that pseudomentalizing is more difficult to detect in the BRFI compared to the AAI due to its short and compact nature, which leaves less space to reflect on oneself and relationships.

At the same time, the RF level in the HC group (M = 4.13) was surprisingly low. However, it still goes along with previous studies which also found a mean RF below 5 (which is generally considered the average rating in a healthy population [18]) in non-clinical samples (e.g [28, 36]. for a use of the AAI and [51, 52] for a use of the BRFI).

The two study groups also differed significantly regarding self-assessed mentalizing (MZQ total score) with the higher scores in the patient group indicating a lower RF level. Interestingly, the between-group differences in self-assessed mentalizing were much more pronounced than the differences in expert-rated mentalizing. Also, when compared with the MZQ total scores found in other mental disorders (e.g. spectrum of 2.6 to 3.4 in a study of Riedl et al. [73] who investigated mentalizing in patients with PTSD, depression and anxiety, adjustment or somatization disorder, respectively), the mean score in the AN group of this study (M = 3.24) ranges at the upper end of the spectrum (indicating worse mentalizing abilities), while their expert rated-RF (M = 3.63) lies in the mid-range when compared to RF scores in other diagnostic groups (e.g. range of 2.7 to 3.9 in a study of Fonagy et al. [74] who investigated mentalizing in patients with depression, anxiety, substance abuse, EDs and personality disorders). On the one hand, this might further validate the assumption of a slight distortion of the patients’ actual RF-BRFI due to pseudomentalizing.

Another possible explanation for the differences in RF-measures could be that even though both BRFI and MZQ tap into various dimensions of mentalizing [22], the MZQ is most commonly regarded as a measure which predominantly assesses *self*-focused mentalizing (e.g [75, 76]). In patients with AN (and other ED) empirical evidence indicates that mentalizing with regard to the self might be particularly impaired [25, 28, 77]. These findings are also in line with the general assumption that EDs are best understood as self-disorders. Based on this, the particularly pronounced impairment in self-assessed mentalizing found in the patient sample of this study can be considered in accordance with previous research.

Next, we examined the relation between mentalizing and ED symptoms and ED-related psychological traits, respectively, in the AN group. As expected, we found significant correlations of both mentalizing measures with various scales of the EDI-2 and the EDE-Q. However, these correlations were much more numerous and larger in effect size for self-reported mentalizing than for the observer-rated RF-BRFI. This difference between the two measures of mentalizing is continued when looking at the additional, explorative analyses regarding the association of mentalizing and body experience: A more negative body experience was strongly and in several of its dimensions correlated with the self-assessment of mentalizing abilities, but not with the expert-rating on the RF-Scale.

The overall weak, rather selective and therefore in total hardly convincing correlation of the RF-BRFI with ED symptomatology is in accordance with several previous studies that also found few or no indication of an association between expert-rated (global) RF and severity of ED symptoms (e.g [36, 37]).

Based on the assumption, that the MZQ primarily captures self-focused mentalizing, its consistent and pronounced correlation with various aspects of both ED symptoms and ED-related psychological characteristics is also in line with previous research: Rothschild-Yakar et al. [77] assessed self-focused mentalizing in patients with ED and HCs using both an RF-expert-rating as well as a self-report questionnaire for alexithymia as a proxy measure. While in the total sample both measures of mentalizing the self were significantly and with moderate to large effect sizes correlated with ED-symptoms, the association was not or only marginally significant for mentalizing regarding others (ToM) and general, expert-rated RF, respectively. Similar results were found in a later study [28]. Overall, these empirical findings suggest, that deficits in self-focused mentalizing might be of particular relevance to the severity of AN and other EDs. Furthermore, they align with the theoretical conceptualization of EDs as self-disorders. Within the framework of the mentalizing model, it could be argued, that a particularly weak and incoherent sense of self and identity might involve a more pronounced impairment of the self-dimension of mentalizing and at the same time predispose to a greater severity of ED-symptoms and related psychological traits.

Another aim of this study was to investigate the relation between mentalizing and general psychopathology. Regarding personality functioning, in accordance with our hypothesis lower self-reported mentalizing abilities in the MZQ were significantly and with a large effect size correlated with more severe impairment in personality functioning (OPD-SQS). No such association was found with regard to the expert-rating of RF. The later finding is in contrast to a study by Zettl et al. [38], who found the RF-BRFI and the OPD-SQS total score to be correlated, even though the effect size was small after controlling for symptom severity. In general, the conceptual overlap between mentalizing and personality functioning is well established. The correlation of both constructs was demonstrated in several studies [38–41] and the findings in the present study concerning the strong association of the MZQ and the OPD-SQS (as *particular* measures of both constructs) are also in accordance with previous research [39]. Interestingly, the strength of the association between these two self-report measures for mentalizing and personality functioning considerably surpasses the effect sizes found for other, interview-based measures of both constructs [40, 41].

Based on previous research that indicated that the impairments in mentalizing abilities of patients with AN might be associated with their low bodyweight [12, 78] as well as the known impact of starvation on cognitive functions [79], we expected an association between the measures of mentalizing and the patients’ BMI. However, we found no association between low body weight and mentalizing. Although this is in accordance with other studies [36, 80], it should be considered that our sample was not ideal for such an analysis: Due to the inclusion of outpatients and patients with atypical AN, the BMI of many patients was comparably high. However, it should also be noted that BMI as a single parameter is not necessarily directly related to the acute degree of undernutrition or malnutrition. Due to an unbalanced diet or a particularly rapid weight loss, individuals with atypical AN may show malnutrition and deficiencies in vitamins and key nutrients, which increases their risk of medical instability, even though they present with a ‘normal’ BMI [81, 82].

Furthermore, we found no significant association of depressive symptoms with either of the mentalizing measures. For the RF-BRFI, this is in accordance with previous research: Several studies found no correlation between expert-rated RF and the severity of depressive symptomatology in samples of patients with depression [43, 83]. However, greater impairment in mentalizing as measured with the MZQ was quite consistently found to be associated with more severe depressive symptoms (e.g [84, 85]). In our sample, a medium effect size of correlation between MZQ and PHQ-9 was found, even though it did not turn out to be significant which is possibly due to the small sample size. This again may indicate a difference within the construct of mentalizing measured with the BRFI and the MZQ as discussed above. Similarily, there was no significant correlation between the severity of somatic symptoms, as measured with the PHQ-15, and either of the mentalizing measures. While we found no association between symptoms of anxiety and expert-rated RF, the GAD-7 correlated significantly and with a large effect size with self-reported mentalizing as measured by the MZQ. This is consistent with previous research, which also found a stronger association between anxiety and mentalization when mentalizing was assessed with questionnaires, compared to an assessment with expert-rated interviews or experimental tasks [86]. Also, the discrepancy in the correlation between the GAD-7 and the RF-BRFI and the MZQ total score, respectively, further supports the assumption, that both mentalizing measures cover different aspects of the overarching construct.

Moreover, our explorative analysis yielded significantly lower self-reported mentalizing abilities (i.e. higher scores in the MZQ) among the patients with binge-purge behavior than among those with only restricting behavior. This is in line with previous research suggesting that patients with AN and other EDs might not be homogenous regarding their impairments in the ability to mentalize [33]. However, these subgroup-differences were only found for the MZQ but not the BRFI. At the same time, only the MZQ (and not the BRFI) was strongly associated with the OPD-SQS. Given that there is some evidence that personality functioning might also be more impaired in patients with the binge-purging subtype of AN than in those with the restricting subtype [87], these results might also be regarded as a correlate of a greater personality dysfunction in patients with binge-purge AN.

Finally, we further examined the association of expert-rated and self-assessed mentalizing as an additional analysis and found a significant correlation of the two measures for the whole sample, but not within the individual groups. This indicates that the association found for the RF-BRFI and the MZQ in the whole sample was actually due to the substantial between-group differences in both of the correlated variables. Therefore, our results do *not* suggest that expert-rated and self-assessed mentalizing were correlated in the present sample. The finding of the correlation for the whole sample is in accordance with a study of Andreas et al. [51] who were able to demonstrate that participants with an above-average RF-level in an interview-based assessment (AAI and BRFI) also showed significantly better self-assessed mentalizing in the MZQ than participants who were assigned a below-average RF score in the expert-rating. However, they did not regard the clinical and the non-clinical group separately. Other studies that investigated the association of an expert-rated and a self-report instrument for parental mentalizing, offered mixed results regarding the correlation of the two measures [88, 89]. Taken together, it seems possible that observer-rated and self-report measures for mentalizing in general as well as the BRFI and the MZQ in particular tap into slightly different aspects of the overarching construct of mentalizing. Thus, they might best be regarded complementary rather than interchangeable. Their combined use may contribute to a more comprehensive understanding of an individual’s capacity to mentalize, if it can be worked out what exactly they measure. At the same time, this implicates that research results on mentalizing might be difficult to compare or to merge if they are based on different measures, especially as long as it is not well-defined which measure captures which aspects of the overall construct.

There are several important limitations to the present study. First, the sample size was rather small and, thus, the statistical power was limited. Especially the results of the explorative analyses should be interpreted with caution. Furthermore, since this study only included adult women, the findings cannot be generalized to other patient groups such as male, transgender or adolescent individuals. Future studies using larger and more diverse samples might allow for a more differentiated assessment of mentalizing among subgroups of patients with AN.

Secondly, exclusion criteria in the control group were assessed only on the basis of self-report questionnaires (i.e. SEED and PHQ) and direct questioning about past or current diagnoses. In order to definitively rule out past or current psychiatric problems, a diagnostic interview by trained clinicians would have been necessary. In addition, both the patient group and the HC group self-reported their current BMI. For a more reliable assessment, it would have been necessary to measure the height and weight of all participants on site.

Thirdly, the majority of patients included in the present study had already received psychotherapeutic treatment for AN which might have improved their ability to mentalize [15, 90]. Future studies might therefore need to employ different recruitment strategies to allow for the assessment of mentalizing abilities in untreated patients.

Fourthly, it was difficult to assess a possible association between impairment in mentalizing and low bodyweight due to the specific composition of the sample. A relevant number of individuals diagnosed with AN had a normal weight, leaving too few cases with a BMI < 17.5 kg/m^2^, resulting in a power problem. Larger studies with a sufficient number of patients in all BMI categories (especially including those who are severely underweight) are required to assess the effect of starvation on mentalizing.

Finally, another important limitation lies in the study’s cross-sectional design. Both mentalizing abilities as well as symptom severity were assessed only at one point in time. Therefore, we could not say whether the deficits found in the patients’ capacity to mentalize represent a state or rather a trait-like characteristic. Future studies with a longitudinal design are necessary to show whether RF changes over time, with weight gain and during the course of a treatment as well as if improvement in RF relates to a reduction of symptom severity and an overall better outcome. To address these important questions, prospective studies are needed that involve a repeated assessment of mentalizing abilities during the therapy process as well as after recovery. Since the iterated use of the same interview protocol might be neither feasible nor reasonable to monitor changes in RF over time, different assessment methods such as the evaluation of mentalizing abilities during psychotherapeutic sessions (In-session RF) might be useful in this context [91].

## Conclusions

Patients with AN showed a lower capacity to mentalize compared to HCs, suggesting that mentalization abilities should be taken into account in the treatment of AN.

A self-report measure of RF showed correlations with several aspects of general and ED-related psychopathology, while an interview-based measure did not. Observer-rated and self-report measures for RF may therefore cover different aspects of the mentalizing construct. A better understanding of which aspects of mentalizing are assessed by different RF-measures is necessary to further clarify the relationship between an impairment in mentalizing abilities and ED-related psychopathology.

## Data Availability

The datasets used and/or analysed during the current study are available from the corresponding author on reasonable request.

## Abbreviations

AN: Anorexia nervosa
BMI: Body Mass Index
BN: Bulimia nervosa
BRFI: Brief Reflective Functioning Interview
HC: Healthy control
ED: Eating disorder
EDE: Eating Disorder Inventory
EDE-Q: Eating Disorder Inventory-Questionnaire
EDI: Eating Disorder Inventory
min: Minutes
MZQ: Mentalization Questionnaire
PHQ: Patient Health Questionnaire
RF: Reflective Functioning
sec: Seconds
ToM: Theory of Mind
